# [Retracted] Mst1 regulates non-small cell lung cancer A549 cell apoptosis by inducing mitochondrial damage via ROCK1/F-actin pathways

**DOI:** 10.3892/ijo.2024.5663

**Published:** 2024-06-10

**Authors:** Weiqiang Zhang, Keiqiang Liu, Yingxin Pei, Jingbo Ma, Jiang Tan, Jing Zhao

Int J Oncol 53: 2409-2422, 2018; DOI: 10.3892/ijo.2018.4586

Subsequently to the publication of the above article, an interested reader drew to the authors' attention that certain of the EdU assay data shown in Fig. 7E on p. 2418 had already appeared in different form in a previously published paper written by different authors at different research institutes.

Owing to the fact that the contentious data in the above article had already been published prior to its submission to *International Journal of Oncology*, the Editor has decided that this paper should be retracted from the Journal. The authors were asked for an explanation to account for these concerns, but the Editorial Office did not receive a reply. The Editor apologizes to the readership for any inconvenience caused.

